# Sarcopenia with postoperative outcomes in patients undergoing pancreaticoduodenectomy

**DOI:** 10.6026/973206300220066

**Published:** 2026-01-31

**Authors:** Nisha B Jain, Javid Ahmad Peer, Ishfaq Ahmad Gilkar, Anil Mehta, Varun Dogra, Umer Mushtaq, Seema Gafurjiwala

**Affiliations:** 1 Department of Surgery, SKS Hospital Medical College and Research Centre, Mathura, Sir gangaram Hospital, New Delhi, India; 2 Department of Surgical Gastroenterology, Gangaram Hospital, Delhi, India; 3 Department of Surgical Oncology, Sher-i-Kashmir Institute of Medical Sciences, Jammu, India; 4 Department of General Surgery, AIIMS, Jammu, India; 5 Department of Statistics, AIIMS, Jammu, India

**Keywords:** Pancreatic cancer, Sarcopenia, body mass index

## Abstract

The prevalence of sarcopenia in patients undergoing pancreaticoduodenectomy (PD) for pancreatic and periampullary tumors and its impact on postoperative outcomes is highly relevant. Therefore, it is of interest to investigate the prevalence of sarcopenia in patients undergoing pancreaticoduodenectomy (PD) for pancreatic and periampullary tumors. A prospective observational analysis of 50 patients revealed that sarcopenia, assessed through Psoas Muscle Index, Hand Grip Strength and Gait Speed, is a significant predictor of postoperative outcomes. Age did not notably affect sarcopenia prevalence, while factors such as gender, ECOG status and diabetes were identified as key risk factors. Gait speed was found to be the strongest predictor of mortality, emphasizing its potential as a primary diagnostic tool. The identification of sarcopenia as a significant predictor of postoperative outcomes in pancreaticoduodenectomy patients with gait speed emerging as the strongest predictor of mortality, offering a potential tool for preoperative assessment and improved patient management.

## Background:

Sarcopenia, the progressive loss of muscle mass and strength, is a key factor influencing surgical outcomes, particularly in complex procedures like pancreaticoduodenectomy (PD), which is commonly performed for pancreatic, bile duct and duodenal tumors [1]. It is increasingly recognized as a predictor of poor outcomes, including higher complication rates, longer hospital stays and decreased survival. Sarcopenia is common in patients with pancreatic cancer and other periampullary tumors due to cancer cachexia, aging and chronic disease [2]. CT imaging, especially at the L3 vertebral level, is commonly used to diagnose sarcopenia by evaluating cross-sectional muscle mass and strength [3]. Recent studies show that preoperative assessment of sarcopenia helps evaluate risks and guide perioperative care. Sarcopenic patients face higher risks of complications, infections and longer ICU stays, with meta-analysis indicating nearly double the risk of postoperative complications [4]. These patients also experience higher readmission rates and reduced long-term survival, possibly due to limited physiological reserves and immune responses. Integrating sarcopenia assessment into routine PD evaluations could improve recovery and survival. Further research is needed to refine diagnostic criteria and develop standardized management protocols [5]. Sarcopenia, characterized by a progressive decrease in muscle mass and function, represents an important health risk for the elderly as well those with chronic diseases. Assessing sarcopenia is essential for early identification, among others; to optimally targets those at risk and provide the right interventions [6]. The Psoas Muscle Index (PMI). It is a reliable, simple and non-invasive method of quantifying muscle mass utilizing imaging data derived from CT scans. It is a summation of CSA (psoas) at the reference vertebral level multiplied by height (cm^2^/m^2^) [7]. The implementation of the PMI definition is methodical; organized and comprehensive measurements can be performed carefully, which articulates ideal tactics established by physical surroundings technology considerations and clinical importance within this manuscript. Therefore, it is of interest to determine the role of sarcopenia in improving outcomes for PD patients.

## Methodology:

This is a prospective observational analytical study which was conducted in the Department of Surgical Gastroenterology and Liver Transplantation at Sir Ganga Ram Hospital in New Delhi for approximately 1 year and 8 months from December, 2019 to July 2021. Included patients were those with pancreatic or periampullary carcinoma who underwent surgery in this study setting and who did not undergo surgery for emergency or palliative indication, multi-visceral resections or patients contraindicated for major surgery. Applying the inclusion and exclusion factors, the patients ranging from 18 to 75 years were included while the patients with inability or no willingness to assess sarcopenia were excluded. Due to lack of prevalence studies regarding sarcopenia in Indian subjects and time limitation, the study was planned in 50 patients only. Sarcopenia was defined according to muscle mass which is psoas muscle area, muscle strength according to handgrip strength and according to physical performance which is linear gait speed. Sarcopenia was defined according to the operational definition proposed by the European Working Group on Sarcopenia - this involves assessment of the reduced muscle mass as compared to psoas muscle index and the demonstration of reduced muscle strength or functional capacity. The proposed hypothesis is to determine frequency of sarcopenia in patient candidates towards PD based on the measure of muscular amount, composition and function as through Primary derived from Psoas Muscle Index, secondary from Hand Grip Strength and tertiary from Gait Speed. This goal seeks to enable a quantitative and qualitative analysis of the levels of sarcopenia in this category of patients using the aforementioned but different parameters that are mutually exclusive to each other and when combined provides a nutshell account of sarcopenia and the health of patients muscles in one way. The detection of Sarcopenia through these distinct standards will aid the knowledge of sarcopenia on surgical outcome in furtherance to the improvement of strategies for better management of the conditions in Oncology. The information was entered in the Microsoft Excel sheet and crosschecked and Analysis using the SPSS statistical software version 24. In this study the values, which are less than 0.05 were considered significant for all the statistical tests used.

## Results:

To analyze the association of sarcopenia with postoperative outcomes after pancreaticoduodenectomy; a total of 50 patients were included in the current study. This included 28 males (56.0%) and 22 females (44.0%). The mean age of the study population was 60.20 years (± 9.44SD) ranging from 37years to 74 years. Most of the patients were in the 51yr - 60yr group (n=18; 36%). The mean BMI was 23.75 (±3.77 SD) with 40% (n=20) of the patients having BMI in the range of 18.5 to 22.9 kg/m^2^. A total of 6.0% (n=3) had BMI ≥30.0 and were considered obese. Among the study population, 72.0% (n=36) of the patients had one or more comorbidities. According to the definition described above all the patients of the study population were divided into the following groups: No sarcopenia, Pre-sarcopenia, sarcopenia and severe Sarcopenia based on whether they had low or normal Psoas Index, Handgrip strength and Gait speed. The study group had 16.0% (n=8) of pre-sarcopenic, 34.0% (n=17) sarcopenic patients and 32.0% (n=16) severe sarcopenic patients. Patients with sarcopenia and severe sarcopenia were considered under the Sarcopenia present group (n=33; 66.0%) and those without sarcopenia (n=13; 43.3%) and pre-sarcopenia were considered under the Sarcopenia absent group (n=17; 34.0%). Diabetes Mellitus is the most common (52.0%; n=26) condition. Comorbidities such as hypertension was noted in 36.0% (n=18), coronary artery disease (CAD) in 8.0% (n=4) and 4.0% patients (n=2) had chronic kidney disease (CKD) and chronic obstructive pulmonary disease (COPD) each. Hypothyroidism was noted in 8.0% of patients (n=4), Remaining 8.0% of patients (n= 4) had other comorbidities including depressive disorder, psoriasis and seronegative arthritis. On analyzing the symptomatology in the study population it was observed that 82.0% (n= 41) patients had presented with pain in the abdomen. 90.0% (n=45) of the patients had obstructive jaundice; which was the most common presentation. 88.0% (n=44) had weight loss owing to malignancy and associated poor appetite. The mean duration of symptoms was 54.80 days (± 39.41) ranging from 15 days to 180 days. Table 1 summarizes the distribution of the presentation. Among the patients with obstructive jaundice (n=45), 48.0% (n=24) of the study patients had undergone preoperative biliary drainage (PBD) before surgery. According to the definition described above all the patients of the study population were divided into the following groups: No sarcopenia, Pre-sarcopenia, sarcopenia and severe Sarcopenia based on whether they had low or normal Psoas Index, Handgrip strength and Gait speed. The study group had 16.0% (n=8) of pre-sarcopenic, 34.0% (n=17) sarcopenic patients and 32.0% (n=16) severe sarcopenic patients. Patients with sarcopenia and severe sarcopenia were considered under the Sarcopenia present group (n=33; 66.0%) and those without sarcopenia (n=13; 43.3%) and pre-sarcopenia were considered under the Sarcopenia absent group (n=17; 34.0%).

There were no significant differences observed in the incidence of sarcopenia in the study population when the basic characters like age, gender, or BMI were compared. The presence or absence of any comorbidities did not significantly affect the incidence of sarcopenia in the study group (p=0.746). Performance status of all patients was assessed preoperatively and it was found to be: ECOG: Grade 0 in 8.0% (n=4), Grade 1 in 42.0% (n=21), Grade 2 in 36.0% (n =18) and Grade 3 in 14.0% (n=7) of patients. On comparing the presence of sarcopenia and ECOG grading in the study population using Chi-squared test, there was a significant correlation between the various groups in terms of distribution of ECOG Category (χ^2^ = 20.053, p = <0.001). Low albumin levels correlated with the presence of sarcopenia (p=0.028). Other pre-op parameters which significantly correlated with sarcopenia were serum total protein (p=0.005), alkaline phosphatase (p=0.011) and gamma- glutamyl transferase (p=0.019). The rest of the pre-op parameters did not significantly influence the presence of sarcopenia in the study cohort. The mean duration of surgery (in minutes) was longer among the sarcopenic group as compared to the non-sarcopenic group (527.18 mins vs. 474.82 mins). However, p-value did not gain statistical significance (Table 2, Table 3). The total number of deaths in the study population during the study period was 5 (10%) (Included 2 female and 3 male patients). All the patients with mortality were found to be sarcopenic. Sarcopenia and mortality were positively related with 15.2% (n=5) of the sarcopenic population having mortality and none (n=0) in the non-sarcopenia patients having mortality. But this correlation did not reach statistical significance (χ^2^ = 2.862, p = 0.152).

## Discussion:

This research assessed the significance of sarcopenia in individuals undergoing pancreaticoduodenectomy. Over half of the participants in the research were discovered to have sarcopenia, a previously unrecorded condition in the Indian population. In this study, 66.0 % of patients undergoing pancreaticoduodenectomy for pancreatic cancer and periampullary tumors were found to have sarcopenia. Research by Wilkinson et al. (2018) [8] indicates that the loss of skeletal muscle mass and strength starts to occur steadily from around the age of forty, with about half of the mass disappearing by the age of eighty. According to reports, muscle mass decreases by 1% to 2% each year after 50 years, while muscle strength decreases by 1.5% to 3% each year after 60 years. In a study of 50 patients undergoing pancreaticoduodenectomy, regional and age-related differences did not appear to affect sarcopenia prevalence, unlike what was reported by the EWGSOP algorithm. In the present study, women showed a greater likelihood of sarcopenia than men, with a relative risk of 1.06 (95% CI: 0.69-1.59), possibly because of the lower muscle mass in females compared to males. The majority of research indicates that sarcopenia was common in women [9]. Tyrovolas *et al.* suggests that inadequate Skeletal Muscle Mass (SMM) impacts BMI by affecting body weight, making a low BMI a predictor of sarcopenia [10]. Nonetheless, in the present research, there was no statistically significant influence of BMI on sarcopenia, Even though there was a weak correlation (p=0.545) several research studies have shown that the patient's performance status is an important factor in predicting postoperative results for cancer patients. The ECOG grading system was utilized to evaluate performance status prior to the surgery. Our research showed a strong connection between ECOG performance status ≥2 and sarcopenia, indicating a relative risk of 2.67 (95%CI: 1.7- 4.76) with a p-value <0.001 deemed significant. The coexistence of diabetes and sarcopenia showed a strong association with a significant p-value of 0.013, similar to a study conducted in Pennsylvania in 2016 by Sousa (2023) *et al.* [11].

Within our study group, 72.0% of participants demonstrate a low Psoas Index, suggesting a high occurrence of reduced muscle mass, especially in the core area. Additionally, 60.0% of people show low Hand Grip Strength, which is an important indicator of overall muscle strength and functional capability. Furthermore, compromised mobility is evident in 42.0% of the population who have a decreased Gait Speed. The results highlight the high occurrence of sarcopenia in the group studied, emphasizing the importance of specific interventions for at-risk individuals to improve muscle mass, strength and functionality. The information indicates a possible area for additional investigation to uncover root causes and create successful tactics for preventing and managing sarcopenia in this group. The research offers a thorough evaluation of how three predictors compare in their ability to predict mortality. The Psoas Index shows a moderate AUROC of 0.658, displaying high specificity at 100% but a low sensitivity at 31%. Hand Grip Strength shows fairly good diagnostic ability (AUROC = 0.698) with equal levels of sensitivity (60%) and specificity (87%). Gait Speed stands out as the most promising predictor, with an impressive AUROC of 0.838, exceptional specificity (100%) and satisfactory sensitivity (62%). Although the Psoas Index is highly specific, its low sensitivity raises doubts about its effectiveness in predicting mortality. These results highlight the significance of taking into account various factors, with Gait Speed standing out as a strong predictor. This result is consistent with the finding of Zhang (2024) *et al.* [9]. Additional validation research is necessary to verify the widespread relevance of these findings and their suitability for various demographics.

## Conclusion:

We show the high prevalence of sarcopenia in patients undergoing pancreaticoduodenectomy for pancreatic cancer and periampullary tumors, with gait speed emerging as a key predictor of mortality. It highlights the importance of addressing muscle-related factors in these patients, with potential clinical applications for gait speed as a primary measure. Further validation studies are needed to refine interventions for sarcopenia prevention and management in this population.

## Figures and Tables

**Table 1:** Summary of Sarcopenia

Sarcopenia	Mean±SD	Min-Max	Frequency (%)
Psoas Index (mm^2^/m^2^)	272.87±97.41	105.80 - 450.80	
**Psoas Index (Low)**			
Psoas Index (Normal)			36 (72.0%)
Hand Grip Strength (Kg)	24.90±7.89	9.80 - 43.60	
Hand Grip Strength (Low)			30 (60.0%)
Hand Grip Strength (Normal)			20 (40.0%)
Gait Speed (m/sec)	0.84±0.22	0.50 - 1.50	
Gait Speed (Low)			21 (42.0%)
Gait Speed (Normal)			29 (58.0%)
**Grades of Sarcopenia**			
No Sarcopenia			9 (18.0%)
Pre-Sarcopenia			8 (16.0%)
Sarcopenia			17 (34.0%)
Severe Sarcopenia			16 (32.0%)
Sarcopenia (Present)			33 (66.0%)

**Table 2:** Association of sarcopenia with pancreaticoduodenectomy complications

**Outcomes**	**Incidence**	**Sarcopenia**		**Chi-Squared Test**	
		Present	Absent	χ^2^	p value
Delayed gastric emptying (DGE)	64.00%	27 (81.8%)	5 (29.4%)	13.375	<0.0013
Clinically relevant POPF (Grade ≥B)	54.00%	20 (60.6%)	7 (41.2%)	1.705	0.1923
Bile leak	14.00%	6 (18.2%)	1 (5.9%)	0.811	0.3982
Surgical site infection	50.00%	18 (54.5%)	7 (41.2%)	0.802	0.3703
Post pancreatectomy hemorrhage	10.00%	4 (12.2%)	0 (0.0%)	4.193	0.0802

**Table 3:** Association between sarcopenia and clavien dindo grade

	**Clavien Dindo Grade**									**Fisher's Exact Test**
Sarcopenia	I	II	IIIa	IIIb	IVa	IVb	V	Total	χ^2^	P Value
Present	3(9.1%)	12 (36.4%)	4 (12.1%)	3 (9.1%)	5(15.1%)	1(3.0%)	5(15.1%)	33(100.0%)		
Absent	10 (58.8%)	6 (35.3%)	1 (5.9%)	0 (0.0 %)	0(0.0%)	0(0.0%)	0(0.0%)	17(100.0%)	18.326	0.004
Total	13 (26.0%)	18 (36.0%)	5 (10.0%)	3 (6.0%)	5(10.0%)	1(2.0%)	5(10.0%)	50 (100.0%)		
